# The maternal–infant microbiome axis as an epigenetic and immunometabolic orchestrator: redefining early-life programming and precision interventions for lifelong women’s and children’s health

**DOI:** 10.1128/iai.00502-25

**Published:** 2025-12-16

**Authors:** Arpita Mukherjee

**Affiliations:** 1 Department of Biochemistry, Shri Venkateshwara University 667521, Gajraula, India; University of California Davis, Davis, California, USA

**Keywords:** maternal–infant microbiome axis, epigenetic programming, immunometabolic regulation, microbial metabolites, neonatal immunity, early-life intervention

## Abstract

The maternal–infant microbiome axis represents a dynamic interface that shapes neonatal immune and metabolic development from the earliest stages of life. Microbial communities from the maternal gut, vaginal tract, and breast milk seed the infant microbiome, influencing chromatin remodeling, transcriptional activity, and immunometabolic programming. Rather than functioning solely as a conduit of microbial inheritance, this axis operates as a regulatory network where microbial metabolites such as short-chain fatty acids and indole derivatives modulate histone acetylation, DNA methylation, and noncoding RNA pathways that calibrate immune tolerance and pathogen defense. Perturbations, including cesarean delivery, perinatal antibiotic exposure, or maternal metabolic disorders, disrupt these processes and are associated with altered immune set points, heightened infection susceptibility, and increased risk of inflammatory and metabolic disease. Multi-omics studies now provide mechanistic insights linking microbial signals to epigenetic regulation of neonatal immune responses, while also exposing important controversies, such as the debated presence of a placental microbiome and the variable efficacy of probiotic interventions. Emerging strategies, including maternal dietary modulation of the microbiome, perinatal microbiota restoration, and development of live biotherapeutics, show promise, but their translational potential remains constrained by limited sample sizes, heterogeneous outcomes, and safety concerns. Framing the maternal–infant microbiome axis as an epigenetic and immunometabolic orchestrator highlights both its therapeutic promise and the need for rigorous mechanistic and clinical evaluation to advance preventive strategies for women’s and children’s health.

## INTRODUCTION

The maternal–infant microbiome axis has emerged as a central determinant of early-life immune and metabolic programming, linking maternal microbial niches with epigenetic remodeling and neonatal immune development. This axis can be conceptualized as an interdependent network encompassing transmitted microbial consortia, microbially derived metabolites, and host gene-regulatory layers, including DNA methylation, histone modifications, and chromatin accessibility, that collectively shape neonatal immune tolerance and pathogen defense (1). Beyond simple vertical transmission, maternal influences act through metabolite-mediated modulation of fetal and neonatal gene expression. Notably, short-chain fatty acids (SCFAs), folate intermediates, and other small molecules cross the placenta or are delivered via breast milk, inducing chromatin remodeling and transcriptional reprogramming in immune progenitors and metabolic tissues (2, 3). Such epigenetic modulation establishes immune–metabolic phenotypes that persist beyond infancy and contribute to long-term susceptibility to allergy, autoimmunity, and infection (4, 5).

Critical developmental windows, including prenatal, perinatal, and early neonatal periods, represent phases of heightened plasticity during which environmental signals can exert durable biological effects (6). For instance, maternal SCFAs have been shown to promote neonatal regulatory T cell differentiation via FOXP3 locus acetylation, while microbial-derived methyl donors can alter hepatic gene expression and metabolic set points (7, 8). These examples illustrate how maternal microbial metabolites influence immune calibration and metabolic resilience, aligning with and extending the developmental origins of health and disease (DOHaD) paradigm (9). Recent metabolomics and transcriptomics studies in germ-free and gnotobiotic models further demonstrate that microbial-derived metabolites such as indoles, aryl sulfates, and SCFAs reach fetal tissues and correlate with immune-related gene expression, reinforcing the concept that maternal microbial status directly shapes fetal epigenetic and immune programming (10, 11). Human cohort investigations are beginning to validate these findings, linking maternal dietary fiber intake and microbial metabolite production with neonatal immune outcomes, although long-term trajectories remain incompletely mapped (12). Despite these advances, major knowledge gaps remain. The tissue- and lineage-specific epigenetic effects of maternal metabolites are poorly characterized, with most data derived from immune cell subsets, while little is known about metabolic organs such as the liver or adipose tissue. The long-term functional implications into childhood and adulthood are still insufficiently defined, particularly in human populations. Furthermore, while maternal dietary modulation, probiotics, and live biotherapeutics show translational potential, their efficacy and safety remain inconsistent, with heterogeneous results across trials.

This mini-review synthesizes current evidence by focusing on (i) maternal microbial metabolites that traverse to the fetus or neonate; (ii) mechanisms by which these metabolites enact epigenetic modifications in neonatal immune and metabolic tissues; (iii) functional links between epigenetic patterning and neonatal immunity; and (iv) disruptions that perturb this axis. By integrating mechanistic insights with translational perspectives, this review highlights the maternal–infant microbiome as an epigenetic and immunometabolic orchestrator, while emphasizing the need for balanced appraisal of evidence and careful evaluation of intervention strategies.

## MATERNAL MICROBIAL NICHES AND VERTICAL TRANSMISSION

The maternal body harbors diverse microbial consortia across distinct anatomical niches, each undergoing pregnancy-associated restructuring that contributes to neonatal colonization and immune programming. The vaginal microbiome is most directly implicated in vertical microbial transfer during delivery. In healthy pregnancies, this niche is typically dominated by *Lactobacillus* spp., which maintain acidic pH and provide antimicrobial defense, though compositional shifts near term may facilitate neonatal gut priming. Perturbations in vaginal communities have been linked to altered neonatal microbial assembly and differential risks of atopy and immune dysregulation (13, 14). The maternal gut microbiome also undergoes marked reorganization during late gestation, characterized by enrichment of Proteobacteria and Actinobacteria with expanded functional capacity for energy harvest and immunomodulation. Experimental models suggest that these changes influence maternal immune tolerance and placental development, in part through the production of metabolites such as SCFAs and indole derivatives that can cross the placenta and affect fetal epigenetic programming (15).

Breast milk represents another critical vertical transmission vector, seeding the infant gut with *Streptococcus, Staphylococcus,* and *Bifidobacterium* and delivering immune-active molecules, including secretory IgA, lactoferrin, and antimicrobial peptides. These components collectively guide neonatal mucosal immunity, and variation in milk microbiota has been associated with infant growth and infection susceptibility (16, 17). By contrast, the concept of a placental microbiome remains one of the most debated issues in perinatal microbiology. Early reports of low-biomass bacterial DNA signatures have largely been attributed to contamination, and current consensus suggests that a stable, functional placental microbiome is unlikely in healthy pregnancies. However, transient microbial or microbial product translocation under pathological states cannot be excluded (18).

**Fig 1:**
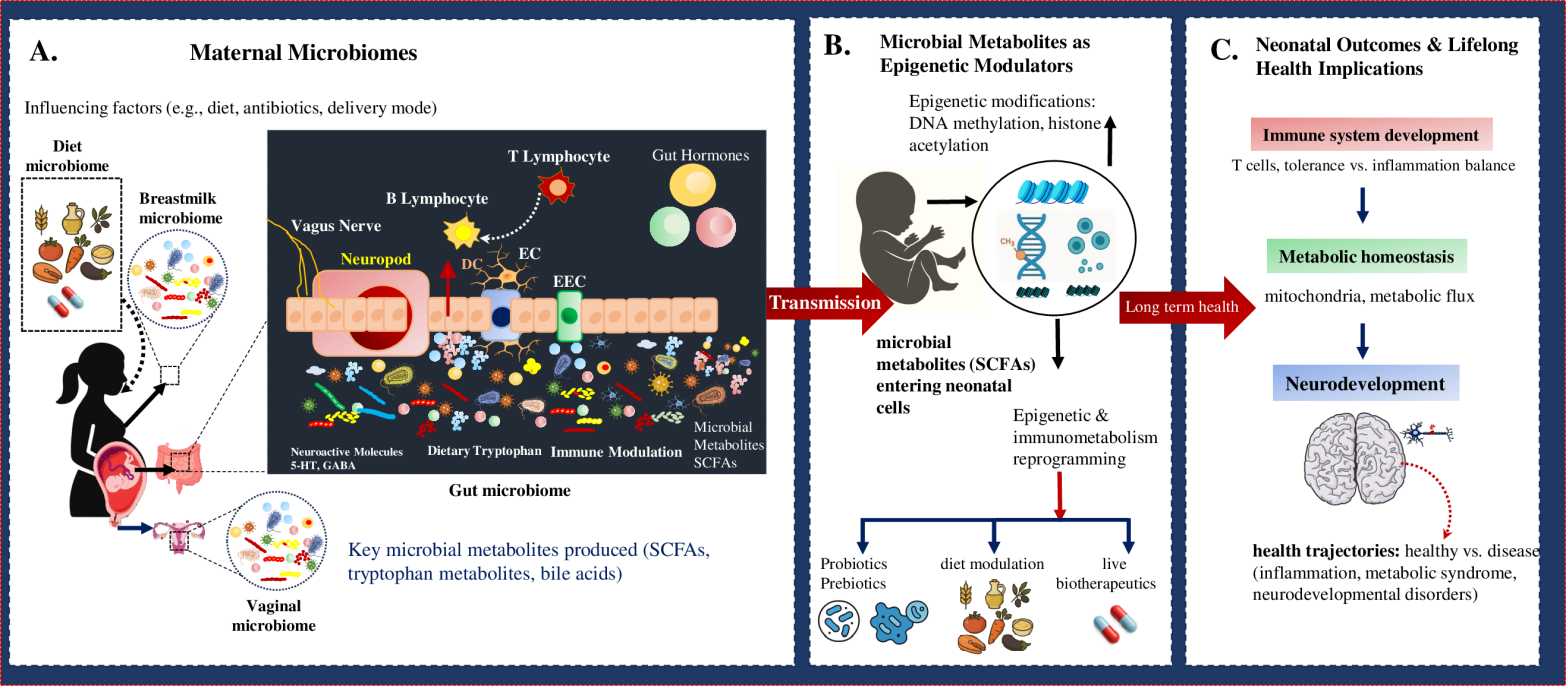
Maternal–infant microbiome axis as a determinant of early-life programming and long-term health trajectories. (**A**) Maternal microbial ecosystems, including gut, vaginal tract, breastmilk, and diet-derived microbiomes, constitute interconnected reservoirs that influence metabolite generation, immune signaling, and neuroendocrine crosstalk during pregnancy and lactation. (**B**) Microbially derived metabolites such as short-chain fatty acids, tryptophan derivatives, and bile acids are vertically transmitted to the neonate, where they act as epigenetic effectors, inducing DNA methylation and histone acetylation changes that drive immunometabolic reprogramming. (**C**) These molecular adaptations shape neonatal immune maturation, metabolic homeostasis, and neurodevelopmental patterning, collectively determining lifelong susceptibility or resilience to inflammatory, metabolic, and neurodevelopmental disorders.

Vertical transmission is further shaped by birth mode, feeding practices, and maternal physiology. Vaginally delivered infants acquire maternal vaginal and perineal taxa, whereas those born via cesarean section (C-section) show delayed microbial diversification and enrichment of skin-associated bacteria, with potential downstream effects on immune maturation (19). Breastfeeding reinforces maternal microbial imprinting through direct bacterial transfer and provision of selective substrates such as human milk oligosaccharides that promote *Bifidobacterium* expansion. Maternal diet and environmental exposures also shape the microbial landscape available for transfer, underscoring the multifactorial nature of maternal–infant microbial assembly (18). Together, these findings highlight maternal microbial niches as finely tuned ecosystems that transmit both microbes and immunomodulatory signals to the neonate. While essential for immune education and colonization resistance, these processes are vulnerable to disruption by cesarean delivery, antibiotics, or maternal metabolic perturbations, with potential long-term consequences for infection susceptibility and immune homeostasis. Evidence from gnotobiotic and germ-free models reinforces this causality, showing that maternal microbiota transplantation accelerates maturation of gut-associated lymphoid tissues, enhances regulatory T cell induction, and improves neonatal resistance to enteric pathogens. These experimental data highlight vertical microbial inheritance as not only correlative but also mechanistically essential for early immune priming and infection defense (20, 21). To encapsulate the mechanistic continuum linking maternal microbial reservoirs to neonatal epigenetic and immunometabolic programming, Fig. 1 provides an integrative schematic overview of the maternal–infant microbiome axis, illustrating how microbial transmission and metabolite signaling collectively shape early-life immune, metabolic, and neurodevelopmental outcomes.

## EPIGENETIC IMPRINTING VIA THE MATERNAL–INFANT MICROBIOME AXIS

The maternal–infant microbiome axis exerts profound influence on the developmental epigenome, integrating microbial metabolite signaling with chromatin remodeling, DNA methylation landscapes, and noncoding RNA networks. This crosstalk orchestrates immune tolerance, metabolic programming, and neurodevelopment during finite windows of heightened plasticity in early life. These processes are pivotal for neonatal adaptation and may establish heritable molecular imprints that extend across generations, reframing the DOHaD paradigm in a microbiome-centric context (22).

### Histone modifications and SCFA-mediated chromatin accessibility

Short-chain fatty acids, notably acetate, propionate, and butyrate, produced by maternal gut microbiota and transferred via the placenta or breast milk, act as histone deacetylase (HDAC) inhibitors. Butyrate enhances H3/H4 acetylation at immune-regulatory loci, promoting transcriptional programs that facilitate T regulatory cell differentiation and metabolic homeostasis (23). In parallel, SCFA-dependent modulation of metabolic regulators such as PPARγ and AMPK integrates nutrient sensing with epigenetic programming (24). In murine models, maternal fiber supplementation elevates SCFAs and epigenetically primes offspring against allergic airway disease (25), demonstrating translational relevance. Beyond acetylation, SCFAs influence histone crotonylation, a mark enriched at active loci associated with lineage specification (26). Such findings highlight a broader metabolic–epigenetic interface in which maternal metabolites fine-tune chromatin states governing neonatal immunity.

### DNA methylation: methyl donors and DNMT activity

DNA methylation patterns are shaped by the availability of one-carbon donors (e.g., folate, betaine, and choline) and the activity of DNA methyltransferases (DNMTs). Microbiome-derived metabolites contribute directly to this pool (27), while SCFAs and tryptophan derivatives signal via GPR41/43 and aryl hydrocarbon receptor (AhR) pathways to modulate DNMT expression (28). Maternal dysbiosis driven by a high-fat diet has been linked to hypermethylation of leptin and adiponectin genes in offspring, predisposing to obesity and insulin resistance (29). Conversely, perinatal probiotic supplementation has normalized methylation at immune checkpoint loci such as PD-1 and CTLA-4, suggesting potential recalibration of tolerance thresholds (30).

### Noncoding RNA regulation

Microbial metabolites also regulate noncoding RNAs. MicroRNAs such as miR-155 and miR-146a, responsive to microbial diversity and SCFAs, influence inflammatory set points in neonates (31). Long noncoding RNAs (lncRNAs), including HOTAIR and MALAT1, implicated in chromatin remodeling and immune gene regulation, are similarly modulated by maternal microbial status (32). These findings suggest that ncRNA networks function as sensitive mediators linking maternal microbial signals to persistent transcriptional landscapes in offspring, with potential as biomarkers of disrupted programming.

### Windows of epigenetic plasticity

Prenatal and immediate postnatal periods constitute windows of heightened susceptibility, characterized by active chromatin remodeling and immature methylation maintenance systems. During these phases, maternal microbiome signals coincide with immune tolerance calibration and hypothalamic–pituitary–adrenal (HPA) axis maturation. Perturbations such as antibiotics, cesarean delivery, or maternal metabolic inflammation may therefore inscribe maladaptive marks with long-lasting consequences (33, 34).

### Transgenerational epigenetic inheritance

Emerging evidence suggests that microbiome-influenced epigenetic modifications may be transmitted across generations, extending the scope of developmental programming beyond the immediate mother–child dyad. In animal models, alterations in maternal microbiota induced by dietary or environmental stressors have been shown to produce persistent DNA methylation and histone modification patterns detectable in the F2 generation, independent of direct microbial transmission (35). Mechanistically, such inheritance may involve the transfer of epigenetically modified germ cells, persistent circulating metabolites, or early-life conditioning of maternal immune and metabolic networks that influence subsequent pregnancies. Recent studies provide further mechanistic support: SCFAs have been demonstrated to exert epigenetic regulation not only in neonatal immune pathways but also in chronic inflammatory and metabolic contexts, underscoring their broad capacity to shape developmental and disease trajectories (36). Complementary *in vitro* and *in vivo* models confirm that butyrate and other SCFAs actively remodel the epigenetic landscape of developing immune tissues, thereby bridging maternal microbial metabolism with long-term offspring health outcomes (37). This transgenerational perspective reframes the maternal–infant microbiome axis as a vector not only for intergenerational health equity but also for the propagation of disease susceptibility across familial lineages.

## IMMUNOMETABOLIC CALIBRATION IN EARLY LIFE

The maternal–infant microbiome axis is a foundational determinant of immunometabolic trajectories, with profound implications for lifelong women’s and children’s health (38). Early-life microbial colonization, shaped by maternal factors, including diet, metabolic status, and gestational microbiota composition, orchestrates the maturation of both immune and metabolic networks in the neonate, effectively calibrating homeostatic set points through epigenetic and metabolite-mediated mechanisms.

### Immune tolerance induction

A central function of early microbial exposure is the establishment of immune tolerance. Commensal-derived signals, particularly SCFAs such as butyrate, propionate, and acetate, promote the expansion and functional stabilization of FOXP3^+^ regulatory T cells (Tregs) within the neonatal gut-associated lymphoid tissue (39). This Treg expansion not only enforces mucosal immune tolerance but also mitigates aberrant Th1/Th17 polarization, thereby reducing susceptibility to autoimmune and atopic disorders (40). Maternal microbial metabolites can transplacentally influence fetal thymic development, shaping Treg ontogeny and imprinting tolerance mechanisms that extend beyond the perinatal window. Concurrently, pattern recognition receptor-mediated interactions between microbial ligands and epithelial or dendritic cells facilitate mucosal immune education, enhancing barrier integrity and fine-tuning cytokine networks for context-specific pathogen defense without eliciting chronic inflammation (41).

### Metabolic programming

Beyond immune calibration, the maternal–infant microbiome axis exerts potent effects on neonatal metabolic programming. Microbial-derived SCFAs serve as substrates for histone acetylation and epigenetic modifications in hepatocytes, adipocytes, and pancreatic β-cells, thereby modulating insulin sensitivity, lipid absorption, and energy homeostasis (42). Specific microbial consortia also influence bile acid metabolism, altering farnesoid X receptor (FXR) and Takeda G protein-coupled receptor 5 (TGR5) signaling pathways, which are critical for lipid and glucose handling in early life. Perturbations in these microbial communities, due to antibiotic exposure or dysbiosis, correlate with aberrant metabolic imprinting, predisposing infants to obesity, insulin resistance, and dyslipidemia in later childhood (43).

### Crosstalk between microbial metabolites and immune checkpoints

Emerging evidence highlights intricate bidirectional interactions between microbial metabolites and immune checkpoint pathways. SCFAs and tryptophan-derived indoles modulate the expression of inhibitory receptors such as PD-1 and CTLA-4 on neonatal T cells, thereby aligning immune activation thresholds with metabolic cues (44). This immunometabolic crosstalk facilitates a balanced immune response, preventing excessive inflammation while preserving pathogen-specific defense and represents a key mechanistic axis for precision interventions aimed at reducing early-life immunometabolic dysregulation (45).

### Integration with neurodevelopmental signaling

The early-life microbiome further interfaces with neurodevelopmental pathways, creating a multidimensional immunometabolic–neurological axis. Microbiota-derived metabolites modulate HPA axis activity, influence microglial maturation, and impact synaptogenesis, thereby integrating metabolic and immune signals into neurodevelopmental programming (46). Such coordination ensures adaptive responses to environmental exposures and maternal nutritional inputs. Importantly, this integrative perspective highlights both opportunities and caveats for microbiome-targeted interventions such as probiotics, prebiotics, maternal dietary modulation, or live biotherapeutics, which may fine-tune immune tolerance, metabolic homeostasis, and cognitive development, but whose efficacy remains variable across populations and study designs (47–49).

In summary, immunometabolic calibration in early life represents a dynamic convergence of maternal microbiota, microbial metabolites, immune tolerance mechanisms, metabolic programming, and neurodevelopmental signaling. This integrative paradigm underscores the potential of precision interventions targeting the maternal–infant microbiome axis to optimize lifelong health outcomes, offering actionable strategies to mitigate immune-mediated and metabolic disorders across the lifespan. To contextualize the integrative roles of maternal microbiome-derived metabolites in shaping neonatal epigenetic, immunometabolic, and neurodevelopmental programming, Table 1 summarizes key metabolite classes, their mechanistic effects, functional outcomes, and representative studies across the maternal–infant axis.

**TABLE 1:** Key maternal microbiome-derived metabolites mediating epigenetic and immunometabolic programming in early life^*a*^

Metabolite class	Representative molecules	Epigenetic effects	Immunometabolic functions	Functional outcomes	Representative references
Short-chain fatty acids	Acetate, propionate, butyrate	HDAC inhibition → ↑ histone acetylation (H3/H4); DNA methylation modulation; histone crotonylation	Treg expansion (FOXP3^+^); balanced Th1/Th17 responses; improved insulin sensitivity	Reduced allergy/asthma risk; protection against obesity/insulin resistance	(23–25, 36, 37, 46)
Tryptophan-derived metabolites	Indole-3-aldehyde, kynurenine, serotonin	AhR activation → altered DNMT expression; modulation of lncRNAs/miRNAs	Regulation of PD-1/CTLA-4; mucosal immune homeostasis; modulation of HPA axis	Balanced inflammation; neurodevelopmental programming	(28, 32, 43, 44)
Bile acids	Primary/secondary bile acids; lithocholic acid	Epigenetic regulation via FXR/TGR5-linked chromatin changes	Lipid/glucose metabolism; antimicrobial defense; innate immune modulation	Metabolic homeostasis; reduced dyslipidemia	(40, 41, 45)
Folate and one-carbon metabolites	Folate, choline, betaine, SAM	One-carbon cycle → DNA/ histone methylation	Regulation of adipogenesis, hepatic metabolism, T cell function	Obesity predisposition (maternal dysbiosis/high-fat diet); metabolic reprogramming	(27, 29, 35)
Human milk oligosaccharides	2′-Fucosyllactose, lacto-N-neotetraose	Indirect epigenetic modulation via selective microbial enrichment (e.g., *Bifidobacterium infantis*)	Shaping gut colonization; sIgA coating; energy harvesting	Lower infection risk; improved growth and cognition	(17, 19, 20, 47)

^*a*^ Functional outcomes listed reflect converging evidence from human cohorts, animal models, and *in vitro* studies. While SCFAs and tryptophan metabolites demonstrate consistent epigenetic and immunoregulatory effects, outcomes related to bile acids, folate/one-carbon metabolism, and HMOs remain context-dependent, with inter-individual variability and study design heterogeneity influencing observed effects. Representative references highlight both mechanistic and translational studies but are not exhaustive. Treg, regulatory T cell; AhR, aryl hydrocarbon receptor.

## DISRUPTION OF THE MATERNAL–INFANT MICROBIOME AXIS: ETIOLOGIES AND CONSEQUENCES

The integrity of the maternal–infant microbiome axis is essential for optimal immune and metabolic programming during early life. Perturbations of this axis can result from multiple etiologies, each of which exerts mechanistic effects on microbial colonization, epigenetic modulation, and immunometabolic development, thereby shaping long-term health outcomes (50).

### Birth mode

Mode of delivery is a primary determinant of neonatal microbial acquisition. Vaginal birth exposes the neonate to maternal vaginal and fecal microbiota, facilitating colonization with *Lactobacillus*, *Bifidobacterium*, and other beneficial taxa. In contrast, C-section delivery delays the establishment of these keystone microbes, favoring opportunistic colonizers such as *Staphylococcus* and *Clostridium* species (51). Such alterations have been linked to impaired Treg development, increased Th2 skewing, and heightened susceptibility to allergic and autoimmune conditions.

### Antibiotic exposure

Perinatal antibiotic exposure, whether maternal or neonatal, disrupts microbial diversity and richness, reducing SCFA production and altering bile acid metabolism (52). Even transient dysbiosis during critical windows of immune and metabolic maturation can impair tolerance induction, compromise epithelial barrier integrity, and perturb insulin and lipid signaling, predisposing offspring to obesity, metabolic syndrome, and immune-mediated disorders (53).

### Maternal metabolic disorders

Maternal obesity and gestational diabetes mellitus (GDM) significantly influence the neonatal microbiome through altered maternal gut and placental microbial composition and metabolite availability (54). Infants of mothers with GDM exhibit early dysbiosis characterized by reduced *Bifidobacterium* and increased pro-inflammatory *Enterobacteriaceae*, correlating with impaired glucose homeostasis and epigenetic modifications in metabolic regulatory genes.

### Nutritional imbalance

Maternal macro- and micronutrient deficiencies or excesses disrupt microbial colonization and metabolic programming. Protein or micronutrient deficiencies reduce microbial synthesis of essential metabolites such as SCFAs and tryptophan derivatives, while high-fat or high-sugar diets enrich pathobionts and promote systemic inflammation (55). These changes interact with neonatal epigenetic machinery, affecting lipid metabolism, energy homeostasis, and neurodevelopmental signaling.

### Environmental stressors and pollutants

Prenatal exposure to pollutants, heavy metals, or psychosocial stress modulates maternal microbial composition and immune status, indirectly influencing neonatal colonization and immunometabolic outcomes (56). Stress-induced cortisol elevations and xenobiotic exposure perturb microbial-derived metabolite profiles, with downstream effects on Treg induction, neuroimmune crosstalk, and metabolic set points.

### Long-term risks

Collectively, disruptions of the maternal–infant microbiome axis increase the risk of allergy, asthma, autoimmune disease, obesity, metabolic syndrome, and neurodevelopmental disorders. These perturbations underscore the critical window of early-life microbial programming, emphasizing the need for targeted interventions, including maternal diet optimization, judicious antibiotic use, stress mitigation, and delivery planning to restore microbial homeostasis and promote lifelong health (57–59).

## MULTI-OMICS INSIGHTS INTO MICROBIOME–EPIGENOME–METABOLOME INTERACTIONS

The convergence of next-generation sequencing, high-resolution metabolomics, and epigenomic mapping has transformed our understanding of how the maternal–infant microbiome axis orchestrates immune and metabolic programming in early life. Multi-omics integration enables simultaneous interrogation of microbial community structure, metabolite output, and host chromatin states, thereby providing a systems-level lens into microbiome–host co-regulation (60).

Metagenomic profiling now extends beyond taxonomic resolution to functional gene content, uncovering microbial pathways that generate immunomodulatory metabolites such as SCFAs, bile acids, and tryptophan derivatives. Untargeted metabolomics complements this by capturing the dynamic repertoire of circulating and tissue-localized small molecules, linking maternal microbial activity to neonatal immune and metabolic circuits (61). In parallel, high-throughput epigenomic assays, including DNA methylation arrays, histone modification mapping, and ATAC-seq, have identified loci selectively responsive to microbial metabolites and nutritional cues, clarifying how early-life exposures establish stable gene-regulatory imprints (62).

Beyond single-omic layers, network biology and machine learning approaches have emerged as critical tools for uncovering keystone taxa and metabolite–gene modules with disproportionate influence on host physiology. Correlation-based microbial–metabolite–epigenetic interaction maps highlight hubs that regulate Treg differentiation, glucose–lipid flux, and neuroimmune signaling (63). Systems-level modeling further enables predictive simulations of interventions, integrating maternal diet composition, microbiome dynamics, and epigenetic reprogramming potential. Such frameworks can forecast the impact of perinatal antibiotics, C-section delivery, or targeted probiotic administration on offspring immunometabolic outcomes, facilitating rational design of precision interventions (64).

Importantly, iterative computational–experimental pipelines now refine these predictions, validating key nodes across animal models and human cohorts. This multi-omics synthesis reframes the maternal–infant microbiome axis as a dynamic and tractable regulatory network, offering not only mechanistic clarity but also translational leverage points for maternal dietary optimization, microbiota restoration, and epigenetic mimicry. Collectively, these approaches lay the groundwork for precision medicine strategies aimed at securing life-course immunometabolic resilience and neurodevelopmental health (6, 65).

## PRECISION AND TRANSLATIONAL INTERVENTIONS

Emerging translational research highlights the maternal–infant microbiome axis as a tractable target for precision interventions designed to recalibrate immunometabolic and epigenetic trajectories during critical developmental windows (66).

### Microbiota restoration strategies

Perinatal restoration approaches, such as vaginal seeding and maternal fecal microbiota transplantation (mFMT), aim to reintroduce keystone maternal taxa into neonates delivered by Cesarean section or affected by maternal dysbiosis. Pilot studies suggest that these interventions can enrich *Bifidobacterium* and *Lactobacillus*, enhance short-chain fatty acid production, and expand neonatal regulatory T cells, thereby reinforcing mucosal tolerance and metabolic homeostasis (67). Nonetheless, standardization, donor screening, and long-term safety assessments are essential before widespread clinical adoption.

### Diet–microbiome engineering

Maternal and neonatal nutritional strategies represent complementary levers for precision modulation. Prebiotics such as inulin and human milk oligosaccharides selectively enrich SCFA-producing microbes, while probiotic strains, including *Bifidobacterium longum* subsp*. infantis* and *Lactobacillus rhamnosus*, stabilize gut barrier function and attenuate inflammatory signaling (68). Symbiotic formulations combine these modalities to simultaneously reshape microbial composition and metabolite flux. Mechanistic studies indicate that butyrate and propionate produced under prebiotic enrichment enhance histone acetylation at metabolic loci, while probiotic supplementation recalibrates dendritic cell signaling and immune tolerance pathways.

### Next-generation live biotherapeutics

Beyond conventional probiotics, engineered microbial consortia are being designed to deliver targeted immunomodulatory metabolites such as tryptophan derivatives and SCFAs at physiologically relevant levels. These next-generation live biotherapeutics hold potential to fine-tune Treg differentiation, reinforce epithelial barrier integrity, and modulate immune checkpoint pathways, including PD-1 and CTLA-4 (69).

### Maternal diet optimization

Maternal diet quality, including macronutrient balance, folate-dependent one-carbon metabolism, and micronutrient sufficiency, exerts a decisive influence on microbial metabolite pools and, consequently, on epigenetic programming in the offspring (70). For example, folate and choline act in synergy with microbiome-derived SCFAs to regulate DNA methylation patterns in metabolic and neurodevelopmental genes, shaping long-term health trajectories.

### Translational challenges and future outlook

Despite promising advances, clinical translation is constrained by inter-individual microbiome variability, heterogeneous trial protocols, and incomplete mechanistic mapping. Rigorous randomized controlled trials, harmonized microbial preparations, and integrative biomarker frameworks spanning microbiome, metabolome, and epigenome layers are urgently needed (71). Looking forward, personalized interventions informed by multi-omics profiling may allow stratified deployment of diet, probiotics, or live biotherapeutics to optimize early-life programming and reduce risks of allergy, autoimmunity, and metabolic disease (72) (Table 2)

**TABLE 2:** Precision and translational interventions targeting the maternal–infant microbiome axis^*a*^

Intervention modality	Mechanism of action	Targeted outcomes	Evidence status	Key reference
Vaginal seeding	Transfer of maternal vaginal microbiota to Cesarean- delivered neonates	Partial restoration of early-life gut microbiota; Treg induction; immune tolerance	Pilot human studies; limited sample sizes	(66)
mFMT	Direct transfer of maternal fecal microbiota	Enhanced microbial diversity; restoration of SCFA production; improved immunometabolic balance	Preclinical and early feasibility studies	(67)
Prebiotics (e.g., HMOs, inulin)	Selective enrichment of SCFA-producing commensals	Epigenetic modulation via histone acetylation; metabolic programming; barrier stabilization	RCTs in infants; moderate evidence base	(68)
Probiotics and synbiotics	Live microbial supplementation ± prebiotic substrate	Enhanced immune tolerance; stabilization of metabolic pathways; attenuation of inflammation	Clinical trials; efficacy variable by strain and context	(69)
Next-generation live biotherapeutics	Engineered microbial consortia producing SCFAs and indole derivatives	Targeted Treg expansion; checkpoint receptor modulation (PD-1, CTLA-4); barrier integrity	Preclinical and proof-of-concept studies	(70)
Maternal diet optimization	Balanced macronutrients; adequate methyl donors and micronutrients	Metabolite-driven DNA methylation and histone modification; support of neurodevelopmental pathways	Observational and interventional studies; emerging evidence	(71)

^*a*^ Summarizes precision interventions targeting the maternal–infant microbiome axis. Strategies range from microbial restoration (vaginal seeding, mFMT) to nutritional modulation (prebiotics, probiotics, synbiotics, and maternal diet) and next-generation live biotherapeutics. These approaches converge on restoring keystone taxa, enhancing SCFA production, and shaping epigenetic immune programming, with evidence spanning pilot studies to preclinical and early clinical trials.

## FUTURE DIRECTIONS

The maternal–infant microbiome axis represents a critical frontier for infection and immunity research, yet major mechanistic gaps remain. Despite compelling associations, causal pathways linking early microbial colonization to neonatal pathogen defense and immune priming are incompletely defined (73). For example, the extent to which specific taxa and their metabolites shape neonatal Treg induction, Th17 restraint, or mucosal barrier resilience during pathogen challenge remains unresolved. Rigorous gnotobiotic and human longitudinal studies are needed to delineate how microbial and epigenetic cues establish durable immune set points (74). Equally pressing is the identification of robust biomarkers to stratify maternal–infant dyads at risk. Candidate indicators include microbiome-derived metabolites (SCFAs and bile acids), epigenetic signatures (DNA methylation of immune loci), and immune profiles (Treg/Th17 ratios and neonatal cytokine fingerprints) (75). Integration of such biomarkers with maternal metabolic and environmental data could refine risk prediction and guide precision interventions. Future work must also critically assess translational strategies such as vaginal seeding, maternal FMT, or engineered probiotics not only for efficacy but also for safety in infection-prone neonates. Addressing these gaps will enable a mechanistic, biomarker-informed framework for optimizing neonatal immune education and resilience against infection while minimizing long-term risks of immune-mediated disease.

### Conclusion

The maternal–infant microbiome axis is increasingly recognized as a master regulator of early-life immunometabolic, epigenetic, and neurodevelopmental trajectories. Microbial colonization, metabolite-driven signaling, and epigenetic remodeling converge to establish immune tolerance, metabolic set points, and cognitive foundations that persist across the life course. Disruption of this finely tuned system through Cesarean delivery, perinatal antibiotics, maternal metabolic disorders, or adverse nutritional and environmental exposures alters microbial composition and function, heightening risk for allergy, autoimmunity, obesity, and neurodevelopmental impairment. Emerging multi-omics approaches integrating metagenomic, metabolomic, and epigenomic data, supported by systems biology modeling, are beginning to map keystone taxa, metabolites, and regulatory loci that underpin these processes. Such insights provide a rationale for precision interventions, including perinatal microbial restoration, maternal nutritional optimization, and next-generation live biotherapeutics. However, robust clinical evidence remains limited, and safety, inter-individual variability, and long-term efficacy demand critical evaluation. Looking forward, longitudinal and multi-generational studies, coupled with biomarker-guided stratification, will be essential to translate mechanistic insights into actionable prevention strategies. Strategic, evidence-based modulation of the maternal–infant microbiome axis holds promise to reduce the burden of immune, metabolic, and neurodevelopmental disorders, advancing the field of maternal and child health toward precision prevention.
